# Editorial of Special Issue “Molecular Mechanisms of Allergy and Asthma 2.0”

**DOI:** 10.3390/ijms241411310

**Published:** 2023-07-11

**Authors:** Daniel P. Potaczek

**Affiliations:** 1 Translational Inflammation Research Division & Core Facility for Single Cell Multiomics, Medical Faculty, Philipps University of Marburg, 35032 Marburg, Germany; potaczek@staff.uni-marburg.de; 2 Center for Infection and Genomics of the Lung (CIGL), Universities of Gießen and Marburg Lung Center (UGMLC), 35392 Gießen, Germany; 3 Bioscientia MVZ Labor Mittelhessen GmbH, 35394 Gießen, Germany

Similarly to the previous Special Issue entitled “Molecular Mechanisms of Allergy and Asthma” [1], this Special Issue aggregates several high-quality original and review articles written by renowned researchers. Not surprisingly, at this time, the multidimensional character of molecular allergology as a field, including both clinics as well as the research approaches, resulted in high thematic variability between the articles, which also makes this Special Issue potentially interesting.

Considering their pivotal role in allergies, T cell-related molecular and cellular mechanisms were the focus of four independent investigational works. Perveen et al. [2] showed that, among protein kinase C (PKC) isozymes, only the levels of PKC ζ (PKCζ) correlated with cytokine production in cord blood T cells (CBTC), thus reinforcing the specificity of CBTC PKCζ levels as a biomarker for risk of allergy development and identifying a “window of opportunity”, in which this could be potentially epigenetically “corrected” after birth. Hu et al. [3] demonstrated, in turn, the successful short-chain fatty acid (SCFA)-augmented in vitro generation of human-induced regulatory T cells (iTregs) along with its mechanisms involving, at least in part, histone acetylation changes. Taking the origin of natural SCFAs in human organisms into account, this could stimulate the development of personalized nutritional therapeutic approaches against allergies, autoimmune, and other chronic inflammatory disorders. Bouté and colleagues [4] observed that the co-stimulation of nucleotide-binding oligomerization domain 2 (NOD2) with muramyl di-peptide (MDP) during allergen sensitization did not worsen Th2/Th17-type allergic airway inflammation in a mouse model; however, MDP co-stimulation of allergen-primed dendritic cells (DCs) promoted a Th2/Th17 profile in asthma patients but not in healthy subjects. Considering that NOD2 adjuvants are used in vaccine design to boost immune responses, whereas care needs to be taken in asthmatics, those might be used in non-sensitized individuals. Goretzki et al. [5], investigated in their highly ambitious study the metabolic mechanisms behind the activation and T cell-modulating potential of DCs stimulated with a fusion protein combining Toll-like (TLR) 5 ligand flagellin (FlaA) and major birch pollen allergen Bet v 1 into a single molecule (rFlaA:Betv1). They found that the effector function of rFlaA:Betv1-activated myeloid DCs mainly depended on glycolysis, with fatty acid synthesis also substantially participating in rFlaA:Betv1-mediated secretion of cytokines, production of antimicrobial molecules, as well as modulation of T cell responses.

Several other papers focused on the molecular mechanisms related to mast cells (MCs), the pivotal effector cells behind allergic responses. Ashikari and coworkers [6] identified an aromatic aldehyde salicylaldehyde as a plant essential oil isolate possessing the properties of an effective inhibitor of the IgE-mediated activation of MCs. Moreover, Nagata et al. [7] characterized the mechanisms behind suppressive effects of a flavonoid kaempferol on MC activation, which turned out to be related to downregulation of the high-affinity immunoglobulin E (IgE) receptor (FcεRI) and upregulation of Src homology 2 domain-containing inositol-5-phosphatase 1 (SHIP1) expression. Furthermore, in the highly intriguing and extensive conceptual review, Babina and colleagues [8] presented, analyzed, and discussed the links between skin MCs and the nervous system. In another review article, Jo et al. [9] summarized the current knowledge on the role of the crosstalk between FcεRI and sphingosine signaling in MC activation and allergic Inflammation. Two further original articles focused on IgE or allergen properties and their role in allergy research and diagnostics. Specifically, McCraw and coworkers [10] described generation and characterization of native and sialic acid-deficient IgE in the context of the importance of those antibodies and their glycosylation in allergo-oncology. Ferrari et al. [11] presented, in turn, a successful specific-IgE detection approach based on the usage of recombinant mouse allergen.

Furthermore, while Nguyen and colleagues [12] focused in their ambitious investigation on atopic dermatitis (AD), showing that the antimicrobial peptide derived from insulin-like growth factor-binding protein 5 (AMP-IBP5) might ameliorate AD-like inflammation and enhance skin barrier function through the receptor low-density lipoprotein receptor-related protein-1 (LRP1), several other papers targeted molecular mechanisms behind asthma, especially those mediating external and/or environmental influences. Concretely, whereas Jorde et al. [13] comprehensively discussed the role of Staphylococcus aureus and its toxins in the pathogenesis of allergic asthma, Kim [14] addressed the complexity of airway epithelium-related immunity in respiratory viral infection-induced asthma exacerbations. Wieczfinska and Pawliczak [15] in turn investigated signaling pathways and transcription regulation involved in airway remodeling in connection with rhinoviral infection. In addition, Rosenberg and coworkers [16] analyzed the effects of prenatal smoke exposure on microRNA expression in fetal lung and their effects on susceptibility to later development of asthma and allergies, while Kaczyńska et colleagues [17] comprehensively summarized current knowledge on the role of regulatory peptides in asthma.

Finally, Zhernov et al. exhaustively described the molecular mechanisms behind eosinophilic esophagitis [18] and scombroid food poisoning [19].

It is with great pleasure that I am presenting the articles included in this Special Issue to the asthma and allergy research community.
